# Neonicotinoid pesticides severely affect honey bee queens

**DOI:** 10.1038/srep14621

**Published:** 2015-10-13

**Authors:** Geoffrey R. Williams, Aline Troxler, Gina Retschnig, Kaspar Roth, Orlando Yañez, Dave Shutler, Peter Neumann, Laurent Gauthier

**Affiliations:** 1 Institute of Bee Health, Vetsuisse Faculty, University of Bern, 3003 Bern, Switzerland; 2 Agroscope, Swiss Bee Research Centre, 3003 Bern, Switzerland; 3 Department of Biology, Acadia University, Wolfville, Nova Scotia B4P 2R6, Canada; 4 Social Insect Research Group, Department of Zoology & Entomology, University of Pretoria, Pretoria 0028, South Africa

## Abstract

Queen health is crucial to colony survival of social bees. Recently, queen failure has been proposed to be a major driver of managed honey bee colony losses, yet few data exist concerning effects of environmental stressors on queens. Here we demonstrate for the first time that exposure to field-realistic concentrations of neonicotinoid pesticides during development can severely affect queens of western honey bees (*Apis mellifera*). In pesticide-exposed queens, reproductive anatomy (ovaries) and physiology (spermathecal-stored sperm quality and quantity), rather than flight behaviour, were compromised and likely corresponded to reduced queen success (alive and producing worker offspring). This study highlights the detriments of neonicotinoids to queens of environmentally and economically important social bees, and further strengthens the need for stringent risk assessments to safeguard biodiversity and ecosystem services that are vulnerable to these substances.

Bees are vital to global biodiversity and food security through their pollination of plants, including several key crops^1^,^2^. Overwhelming evidence now suggests that numerous wild and managed bee populations are in decline, likely because of multiple simultaneous pressures including invasive parasites, changes to climate, and changing land use^3^,^4^. This has led to concerns over human food security and maintenance of biodiversity. The neonicotinoid class of chemical pesticides has recently received considerable attention because of potential risks it poses to ecosystem functioning and services^5^. Ubiquitously used for management of harmful insects in the last decade, these systemic chemicals persist in the environment, thereby promoting their contact with non-target organisms such as pollinating bees^6^.

Alarmingly, exposure to field-realistic concentrations of neonicotinoids impairs productivity of important social bee pollinators^7^,^8^,^9^ that have, among females, reproductive division of labour between workers and queens. A plethora of literature has demonstrated lethal and sub-lethal effects of neonicotinoid pesticides on social bees in the field and laboratory. These examinations have focused largely on workers (females chiefly responsible for essential colony housekeeping and foraging duties rather than reproduction; their production of haploid offspring is primarily regulated by queen pheromones and other colony conditions^10^,^11^), and to a lesser extent overall colony function^7^,^12^,^13^. The role of queens (primary reproductive females that can produce diploid offspring) in social bee colony survival is indispensable, and relies heavily on *a priori* successful development and successful mating flights that trigger profound molecular, physiological, and behavioural changes^10^,^14^. Previous investigations have observed that bumble bee colonies exposed to neonicotinoids produced fewer gynes (future queens)^9^,^15^ and that honey bee colonies replaced queens more frequently^8^; however, mechanisms responsible for these observations have not been identified. This is remarkable considering anecdotal reports of ‘poor quality queens’ (*i.e.* queen failure) of an important pollinating species, the western honey bee (*Apis mellifera*; hereafter honey bee), throughout the northern hemisphere^16^.

In this study, we hypothesised that exposure to field-realistic concentrations of neonicotinoid pesticides would significantly reduce honey bee queen performance due to possible changes in behaviour, and reproductive anatomy and physiology. To test this, we exposed developing honey bee queens to environmentally-relevant concentrations of the common neonicotinoid pesticides thiamethoxam and clothianidin. Both pesticides are widely applied in global agro-ecosystems^17^ and are accessible to pollinators such as social bees^18^, but are currently subjected to two years of restricted use in the European Union because of concerns over their safety^19^. Upon eclosion, queens were allowed to sexually mature. Flight behaviour was observed daily for 14 days, whereas production of worker offspring was observed weekly for 4 weeks. Surviving queens were sacrificed to examine their reproductive systems.

## Results

### Queen rearing success

No significant difference between treatments was observed for queen rearing success (*i.e.* grafting to emergence) (contingency table 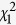 = 0.3, *P *= 0.61). Success was 38.1 ± 9.5% and 44.0 ± 14.6% in the controls and neonicotinoids, respectively (mean ± standard error).

### Mating nucleus colony observations

**Figure 1:**
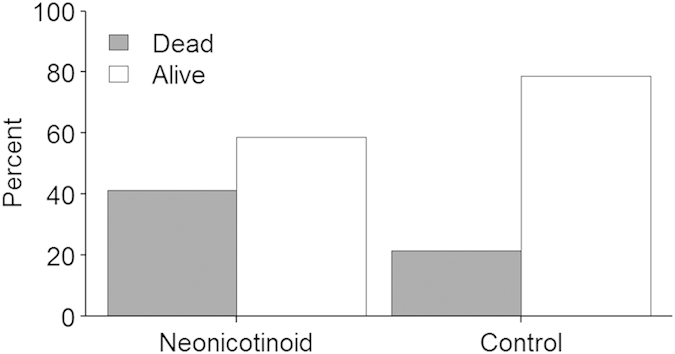
Queen survival after 4 weeks. Percent honey bee queens that were alive after 4 weeks. No significant difference was observed between treatments. *P ≤ 0.1, **P ≤ 0.05, ***P ≤ 0.01 (comparison with Controls).

**Figure 2:**
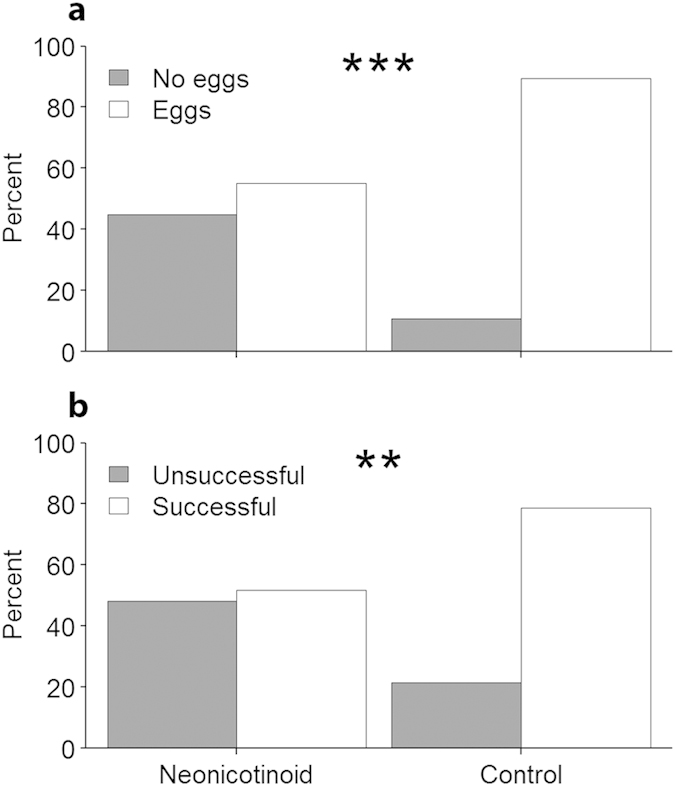
Queen oviposition and survival after 4 weeks. (**a**) Percent of honey bee queens that oviposited (*i.e.* laid worker eggs). **(b**) Percent of honey bee queens that were alive and had produced diploid offspring by the end of the experiment (= Successful). Significant differences between treatments denoted by *P ≤ 0.1, **P ≤ 0.05, ***P ≤ 0.01.

After four weeks post queen emergence, 25% fewer neonicotinoid queens were alive compared to controls (contingency table 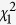 = 2.6, *P *= 0.11; Fig. 1). Regardless of whether they survived to four weeks, 38% fewer neonicotinoid queens produced workers compared to controls (contingency table 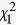 = 8.2, *P *= 0.004; Fig. 2a). Even within our abbreviated observation interval, a significant 34% reduction in success (*i.e.* alive and producing worker offspring) was observed among neonicotinoid-exposed queens compared to controls (contingency table 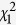 = 4.5, *P *= 0.03; Fig. 2b).

**Figure 3:**
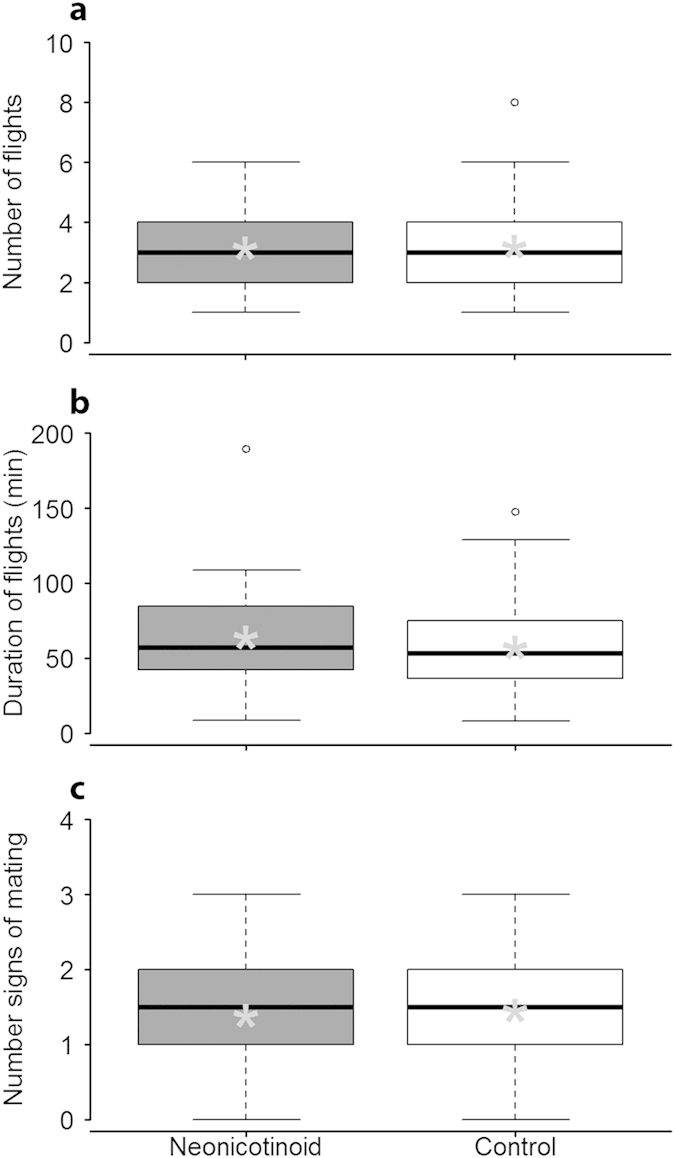
Queen flight over a 4-week interval. (**a**) Number of flights by honey bee queens. **(b)** Total duration of flights by honey bee queens (**c**) Number of signs of mating. Boxplots show inter- quartile range (box), median (black line within interquartile range), means (grey asterisk), data range (dashed vertical lines), and outliers (open dots). No significant difference was observed between treatments for any measure. *P ≤ 0.1, **P ≤ 0.05, ***P ≤ 0.01 (comparison with Controls).

**Figure 4:**
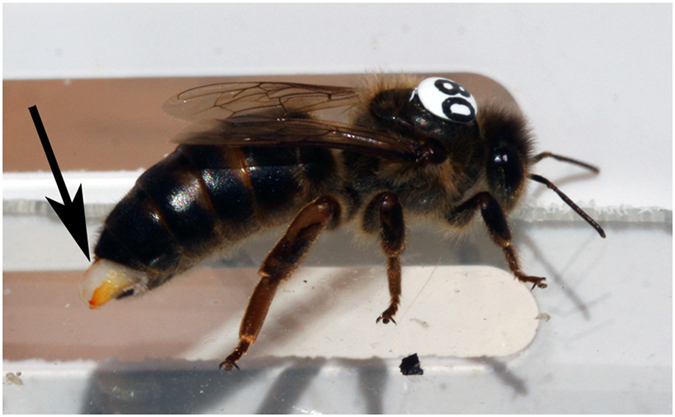
A marked queen returning to the entrance of a baby mating nucleus hive during experimental observations; arrow denotes mating sign (remnants of a male’s everted endophallus protruding from the queen’s vagina^20^).

No difference between treatments was observed for any measured queen flight parameter; both sets of queens undertook similar numbers (Kruskal Wallis 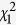 = 0.1, *P *= 0.99; Fig. 3a) and durations (mixed model with queen as a random factor, *F*_1,174 _= 0.2, *P *= 0.67; Fig. 3b) of flights, and had comparable signs of mating (*i.e.* remnants of a male’s everted endophallus inserted into the opening of the returning queen’s reproductive tract^20^) (contingency table 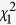 = 1.9, *P *= 0.17; Fig. 3c, Fig. 4).

### Queen dissections and laboratory measurements

**Figure 5:**
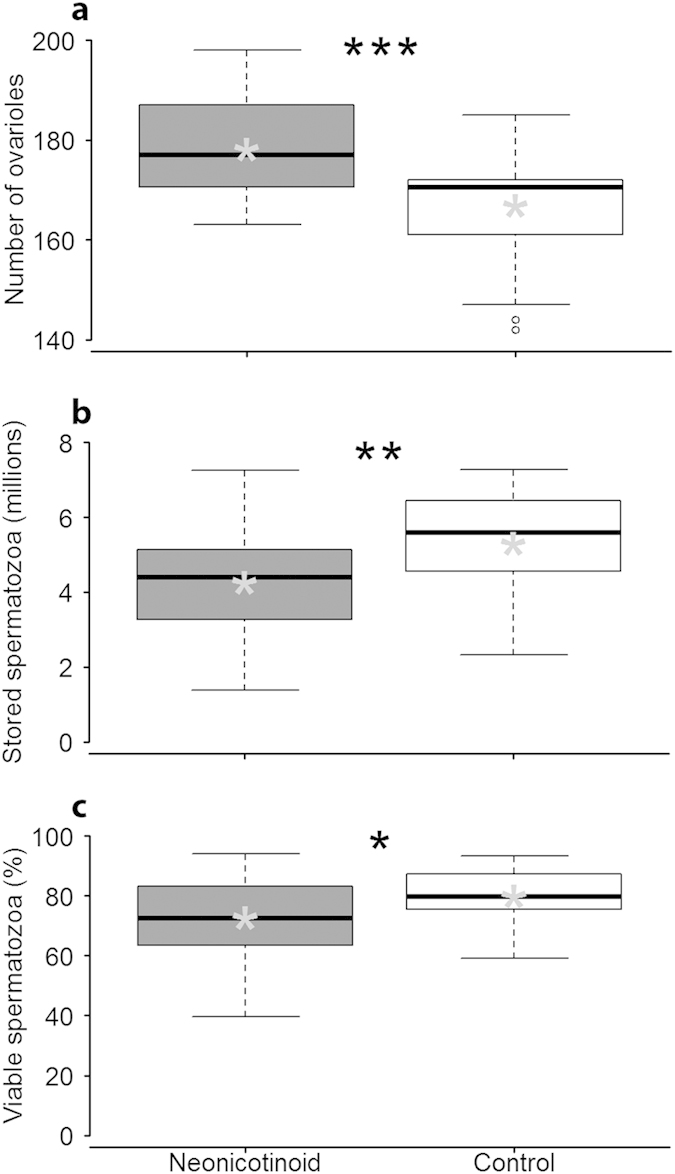
Queen anatomy and physiology after 4 weeks. (**a**) Ovary size, represented by number of ovarioles, of honey bee queens. (**b**) Number of spermatozoa stored in spermathecae of honey bee queens. (**c**) Percent viable spermatozoa stored in spermathecae of honey bee queens. Boxplots show inter- quartile range (box), median (black line within interquartile range), means (grey asterisk), data range (dashed vertical lines), and outliers (open dots). *P ≤ 0.1, **P ≤ 0.05, ***P ≤ 0.01 (comparison with Controls).

For queens surviving the four-week observation period, ovary sizes of those exposed to neonicotinoids were 6.8% larger compared to controls (ANOVA *F*_1,35 _= 9.0, *P *= 0.005; Fig. 5a). Neonicotinoid queens had 20% fewer stored spermatozoa (*F*_1,35 _= 4.8, *P *= 0.03; Fig. 5b) and a 9% lower proportion of living versus dead sperm (*F*_1,35 _= 3.3, *P *= 0.08; Fig. 5c). For the queens that survived and produced worker offspring (*N *= 37), there were no significant correlations among emergence mass, ovariole number, sperm number, or sperm vitality (all |r| < 0.26, all *P *> 0.13). Similarly, no significant differences were observed when queens were separated by treatment (controls: max |r| = 0.24, minimum P = 0.29 (*N *= 22), neonicotinoids: max |r| = 0.21, minimum P = 0.44 (*N *= 15)).

## Discussion

The results demonstrate for the first time possible mechanisms by which exposure to field-realistic concentrations of neonicotinoid pesticides during development can significantly affect queens of a social bee. Increased rates of honey bee queen failure have been reported in recent years^21^. Even within our abbreviated observation interval, we observed significant effects of neonicotinoids on honey bee queen anatomy and physiology, but not behaviour that resulted in reduced success (*i.e.* dead queens or living ones not producing worker offspring). Additionally, we found no significant effect on queen rearing success (proportion of emerged queens) between the treatments, suggesting that there were no lethal effects of pesticide during this stage of queen development. Because honey bees are haplodiploid, wherein males typically result from unfertilised eggs and females (*i.e.* workers or queens) develop from fertilised ones, production of workers confirms successful queen mating^10^. Honey bee queens seldom start to oviposit beyond 3 weeks of emerging^20^, so absence of developing workers in a colony during our 4-week observation period most likely suggests that a queen did not mate or was for some other reason unable to lay fertilised eggs^10^.

Honey bee queens are highly polyandrous, and normally embark on a series of mating flights within 14 days of emerging from their cells during which they should be fertilised with a sufficient number of spermatozoa to last their lifetime; they rarely leave the colony once they start ovipositing^10^. Our study suggests that queen flights were not influenced by neonicotinoid exposure because similar frequencies and lengths were observed compared to controls. This was unexpected because neonicotinoids can negatively affect worker bee flight behaviour^7^,^12^. It is possible that our study investigating queen flights cannot be directly compared to these studies due to differences among investigations regarding female caste (queen versus worker), model species (honey bee versus bumble bee), experimental treatment (neonicotinoids thiamethoxam and clothianidin vs. the neonicotinoid imidacloprid and the pyrethroid λ-cyhalothrin), experimental method (visual observations vs. radio-frequency identification tagging), treatment exposure (colony versus individual), or task measured (mating versus foraging).

Longevity of honey bee queens depends largely on proper development to sexual maturity and appropriate behavioural, anatomical, and physiological changes that occur following successful mating^10^,^14^. Therefore, negative effects on delicate queen reproductive systems that result in abnormal physiology or anatomy, or that impair storage of spermatozoa or oviposition, could result in costly queen replacement by the colony^10^. Surprisingly, we observed ovariole hyperplasia in neonicotinoid-exposed queens compared to controls. Increased ovary size suggests that neonicotinoids can affect development of queen reproductive system; it is unclear how hyperplasia observed here may influence egg production and fertilisation, or may correspond to other anatomical or physiological changes. Furthermore, we observed a significant reduction in the number and quality of stored spermatozoa within queen spermathecae. It is possible that neonicotinoids, due to neuronal hyper-excitation^22^, cause dysfunction of queen physiology and anatomy responsible for transporting and storing newly-received drone spermatozoa during mating. Proper storage of adequate quantities of spermatozoa is crucial to queen survival because a queen is quickly replaced by a colony after depletion of healthy spermatozoa^10^.

Poor queen health is considered an important cause of honey bee colony mortality in North America and Europe^16^,^23^, yet few data can explain these observations over such broad regions^24^,^25^. Considering the widespread use of neonicotinoids in developed countries, our study suggests that these substances are, at least partially, responsible for harming queens and causing population declines of social bee species. Failure of queens exposed to neonicotinoids during development to successfully lay fertilised eggs that subsequently develop into workers or queens is worrisome; both castes are vital to colony survival, particularly when emergency queen replacement is needed. This is especially important for wild social bees that cannot rely on human intervention to mitigate effects of queen failure or colony mortality.

Current regulatory requirements for evaluating safety of pesticides to bees fail to directly address effects on reproduction^26^. This is troubling given the key importance of queens to colony survival and their frailty in adjusting to environmental conditions. Our findings highlight the apparent vulnerability of queen anatomy and physiology to common neonicotinoid pesticides, and demonstrate the need for future studies to identify appropriate measures of queen stress response, including vitellogenin expression^27^. They additionally highlight the general lack of knowledge concerning both lethal and sub-lethal effects of these substances on queen bees, and the importance of proper evaluation of pesticide safety to insect reproduction, particularly for environmentally and economically important social bee species.

## Methods

### Apiary setup

The study was performed in Bern, Switzerland, during May-September 2013 using *A. mellifera carnica* honey bees. Six sister queen experimental colonies were established in early May; each contained typical quantities of adults, immatures, and food (honey and beebread) for the season. Colonies were randomly assigned to either neonicotinoid or control treatments, with each group represented equally.

### Pesticide treatment

Treatments were administered via pollen supplements that were prepared from bee-collected pollen and honey (3:1 by mass, respectively) obtained from non-intensive agricultural areas of Switzerland. Supplements for the neonicotinoid treatment were additionally spiked with 4 ppb thiamethoxam and 1 ppb clothiandin (both Sigma-Aldrich) to represent environmentally relevant concentrations observed in pollen of treated crops^28^,^29^. These amounts were confirmed (4.16 and 0.96 ppb for thiamethoxam and clothianidin, respectively) by the French National Centre for Scientific Research using ultra-high performance liquid chromatography-tandem mass spectrometry (UHPLC-MS/MS). Colonies were each outfitted with a pollen trap prior to administering treatments. This promotes pollen supplement consumption by removing bee-collected pollen from returning foragers. Each colony received 100 g pollen supplement every day for 36 days to ensure that colonies contained young bees exposed to the neonicotinoids during queen rearing; supplements were well-received, but never completely consumed during each feeding period.

### Queen rearing

Queens were produced in experimental colonies using standard honey bee queen-rearing techniques^30^. Briefly, original sister queens were removed from colonies 27 days post initial exposure to create queenless cell-builder nuclei, each composed of 2 food frames and 1 kg brood nest workers. One-day old larvae from each colony were grafted into artificial queen cells and subsequently placed in respective cell-building nuclei overnight. Contents of each cell-building nucleus, including artificial queen cells, were returned to their original experimental mother colony the following day to ensure proper queen development; colonies continued to receive pollen supplements until after queen cell-capping. Prior to emergence, queens were transferred to cages supplied with a food paste (1 part honey: 3 parts powdered sugar by mass) that were maintained in the laboratory in complete darkness at 34.5 °C and 60% humidity^31^. Queen cells were observed every 6 hours starting 11 days post-grafting. Emerged queens were visually inspected, numbered on the dorsal thoracic plate using queen marking numbers, and re-caged with five attendant workers from her mother colony during the expected period of queen emergence (~1 day). Subsequently, each queen was placed in a mating nucleus hive (APIDEA Vertriebs) with 300 g apiculture candy (Südzucker) and 100 g brood nest workers from her original mother colony, and confined for 3 days in darkness at 12 °C to promote colony formation prior to placement outdoors. In total, 29 neonicotinoid and 28 control queens were employed for the ensuing performance measures.

### Mating nucleus colony observations

**Figure 6:**
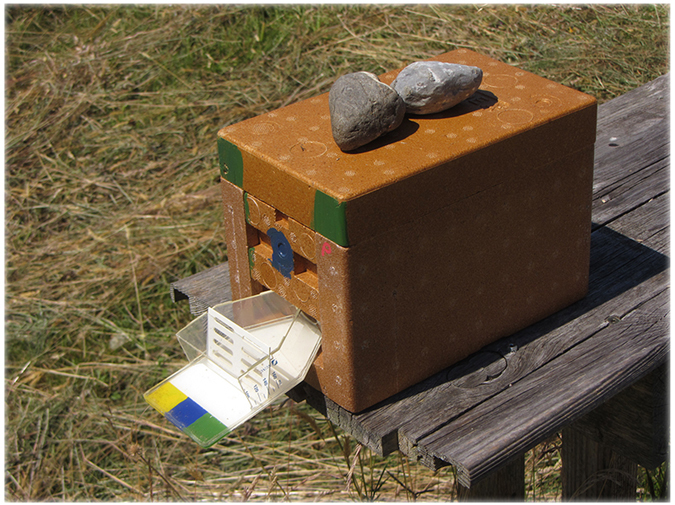
Experimental baby mating nuclei; each nucleus was equipped with a modified entrance consisting of a flat plastic flask and apiculture queen-excluding screen to observe exiting and returning queens without disrupting workers.

Entrances of mating nucleus colonies were observed daily from 11.00–17.00 for 14 days, the typical period of queen flight^10^. Each colony was equipped with an observation landing board constructed using a flat plastic flask (ThermoFisher Scientific) and apiculture queen-excluding screen to document exiting and returning queens without disrupting workers (Fig. 6). Flights by queens were defined as periods away from colonies, including observations on landing boards. After the initial 14-day entrance observation period, presence of queens and developing workers was assessed weekly for an additional 14 days by visually inspecting all frames of each mating nucleus.

### Queen dissections and laboratory measurements

Queens surviving the 4-week mating nucleus observation and assessment period (16 and 22 neonicotinoid and control queens, respectively) were removed from their colonies and anaesthetised using carbon dioxide to allow for inspection of their reproductive anatomy. Spermathecae and ovaries were removed and placed in Kiev buffer^32^ or PBS buffer supplemented with 2% paraformaldehyde, respectively. Numbers of spermatozoa stored in each spermatheca were calculated using a Thoma haemocytometer (ThermoFisher Scientific) using compound microscopy (Model BX41, Olympus)^33^. Viability of spermatozoa in spermathecae was determined using a laboratory kit (Live/Dead^®^ Viability Kit, Life Technologies), wherein a 50-μl aliquot suspension of the spermathecal content was dyed using SYBR-14 and propidium iodide to view 10 fields of view of living and dead spermatozoa, respectively, using fluorescent compound microscopy (Model BX41, Olympus). Number of ovarioles per ovary was determined by real-time counting under stereo microscopy (Model SZX10, Olympus) using a fine needle^34^.

### Statistics

Statistical software was used to perform analyses (SAS 9.3; SAS Institute) and to create figures (R 2.15.3; The R Foundation for Statistical Computing). Comparison of numbers of queens from each treatment that were both alive and producing workers (successful) versus either dead or not producing workers (unsuccessful) was done with contingency table analyses, as were comparisons of numbers of queens alive versus dead, and numbers of queens producing workers versus not. For some flight comparisons, most queens appeared multiple times in the data; to account for pseudoreplication, queen was a random factor in mixed models. For reproductive parameters, non-parametric Kruskal-Wallis tests were used when data were not normally distributed, whereas ANOVAs were used when data were normally distributed^35^,^36^. Correlation analyses (Pearson and Spearman gave qualitatively similar results) were used to evaluate associations among emergence mass, ovariole number, number of spermatozoa, and sperm vitality.

## Additional Information

**How to cite this article**: Williams, G. R. *et al.* Neonicotinoid pesticides severely affect honey bee queens. *Sci. Rep.* **5**, 14621; doi: 10.1038/srep14621 (2015).
